# Effect of probiotic fermented dairy products on incidence of respiratory tract infections: a systematic review and meta-analysis of randomized clinical trials

**DOI:** 10.1186/s12937-021-00718-0

**Published:** 2021-06-28

**Authors:** Kamil Rashidi, Bahman Razi, Mina Darand, Azadeh Dehghani, Parisa Janmohammadi, Shahab Alizadeh

**Affiliations:** 1 Department of Food Sciences and Technology, Faculty of Nutrition and Food Technology, National Nutrition and Food Technology Research Institute, Shahid Beheshti University of Medical Sciences, Tehran, Iran; 2 Department of Hematology and Blood Banking, School of Medicine, Tarbiat Modares University (TMU), Tehran, Iran; 3 Department of Clinical Nutrition and Dietetics, Faculty of Nutrition Sciences and Food Technology, National Nutrition and Food Technology Research Institute, Shahid Beheshti University of Medical Sciences, Tehran, Iran; 4 Nutrition Research Center, Department of Community Nutrition, Faculty of Nutrition and Food Science, Tabriz University of Medical Sciences, Tabriz, Iran; 5 Student Research Committee, Tabriz University of Medical Sciences, Tabriz, Iran; 6 Department of Clinical Nutrition, School of Nutritional Sciences and Dietetics, Tehran University of Medical Sciences (TUMS), Tehran, Iran

**Keywords:** Probiotics: Fermented milk, Fermented dairy products, Respiratory tract infections, Meta-analysis

## Abstract

**Background:**

Previous studies have suggested that the consumption of probiotic fermented dairy products (PFDP) may have a protective effect on respiratory tract infections (RTIs). However, the results of studies are inconclusive. We aimed to systematically investigate the effect of PFDP on RTIs by performing a meta-analysis of randomized controlled trials (RCTs).

**Methods:**

PubMed and Scopus databases were systematically searched up to October 2020 to identify eligible RCTs. Meta-analysis outcomes were risk of incidence of upper (URTIs ) and lower (LRTIs ) respiratory tract infections. A random-effects model was used to pool the relative risks (RR) and corresponding 95 % confidence intervals (CI) for outcomes following conception of PFDP.

**Results:**

A total of 22 RCTs, with a total sample size of 10,190 participants, were included in this meta-analysis. Compared with placebo, consumption of PFDP had a significant protective effect against RTIs in the overall analysis (RR = 0.81, 95 %CI: 0.74 to 0.89) and in children (RR = 0.82, 95 %CI: 0.73 to 0.93), adults (RR = 0.81, 95 %CI: 0.66 to 1.00), and elderly population (RR = 0.78, 95 %CI: 0.61 to 0.98). The significant decreased risk of RTIs was also observed for URTIs (RR = 0.83, 95 %CI: 0.73 to 0.93), while, this effect was marginal for LRTIs (RR = 0.78, 95 %CI: 0.60 to 1.01, *P* = 0.06). The disease-specific analysis showed that PFDP have a protective effect on pneumonia (RR = 0.76, 95 %CI: 0.61 to 0.95) and common cold (RR = 0.68, 95 %CI: 0.49 to 0.96).

**Conclusions:**

Consumption of PFDP is a potential dietary approach for the prevention of RTIs.

**Supplementary Information:**

The online version contains supplementary material available at 10.1186/s12937-021-00718-0.

## Research highlights

- Consumption of PFDP had a significant protective effect against RTIs in all age groups.
- PFDP had a protective effect on pneumonia and common cold.
- The protective effect of PFDP was modified by probiotic genus and type of dairy product used for intervention.

## Background

Acute respiratory infections (RTI, including upper RTI (URTI), e.g., cold, and lower RTI (LRTI), e.g., pneumonia and bronchitis, are a pervasive public health problem in all developed and developing countries, leading to nearly four million deaths annually, with more than 60 deaths per 100,000 population [1]. ARIs are a main public health problem worldwide and contribute to increased morbidity and mortality, as they result in a large number of outpatient visits, hospital admissions, and the widespread administration of antibiotics [2]. These diseases affect all age groups every year and put a heavy burden on the world’s health and economic systems. More than 200 types of viruses have been identified as causing respiratory diseases [3, 4]. As well as, 90 % of deaths because of respiratory infections are reported to happen in patients over 65 years of age [5]. Young children are more likely to get this respiratory infection than adults or children [6, 7]. In most cases, the diseases of the upper respiratory tract are mild to moderate and mostly self-limiting. However, LRTIs-induced pneumonia can be predominantly fatal in children and the elderly or in immunocompromised individuals [4, 8].

The health benefits of fermented milk and dairy products have long been known. The health benefits of dairy products are the consequence of the biologically active ingredients existing in native milk and are also produced in fermented or sour milk products produced by the action of probiotic bacteria [9]. Probiotics have been used as an adjunct to reduce the risks of widespread use of antibiotics such as diarrhea and to prevent infections, including respiratory infections [10]. One reason that probiotics are considered to be the main and important components of the diet to reduce the risk of infectious diseases is due to their functional role in the gastrointestinal tract and intestinal epithelium, as well as their relationship with the function of the immune system and intestinal mucosa [11, 12]. Probiotics are living microorganisms that, if administered in sufficient doses, provide health benefits to the host [13].

Studies show that functional foods from fermented cow’s milk with probiotic strains can well prevent infectious diseases, but the data are still inconsistent [14–19]. Prevention or control of infectious diseases is one of the most promising health benefits of probiotics [20–22]. The useful effects of lactic acid bacteria and cultured milk products have been ascribed to their capability to suppress the growth of pathogens instantly or via the genesis of antibacterial agents [23]. The results of several studies have shown that some probiotics are effective against infections of the gastrointestinal tract and respiratory tract [24]. Furthermore, it has been demonstrated that probiotics have an essential role in extenuating the rate of ARI episodes and antibiotic use [25]. Nonetheless, there are little well-designed individual interventional studies, with contradictory findings, assessing the clinical effects of dairy, mostly for yogurt and milk, supplemented with chosen probiotics against acute RTI.

Considering the potential of probiotic products and the importance of medical nutrition therapy of respiratory tract infections, the current meta-analysis of recently conducted randomized controlled trials (RCTs) aimed to assess the effect of fermented dairy products by probiotics on incidence of respiratory tract infections in children, adults, and elderly.

## Main text

### Methods

#### Search strategy

We followed PRISMA guidelines (Preferred Reporting Items for Systematic Reviews and Meta-Analyses) in the design and reporting of the methods for this systematic review [1]. PubMed and Scopus online databases were searched from inception to October 2020 for RCTs examining the effect of probiotic fermented dairy products on incidence of respiratory tract infections. A different combination of keywords was searched (Supplemental file 1). Where possible, Medical Subject Headings in addition to free-text search terms were used in the search. The search results were limited to English-language publications. In addition, we checked references of retrieved eligible papers and previous review articles in this area to make sure we found all relevant articles.

#### Screening and study selection

First, electronic and manual search results were exported to EndNote software, version X8 (Thomson Reuters) and duplicate publications were eliminated. Selection of eligible controlled trials was carried out independently by two investigators (SA & PJ). Any discrepancies were resolved by consensus. The researchers first assessed the titles and abstracts of studies obtained through preliminary searches, then, independently reviewed the full text of remaining publications.

#### Eligibility criteria

The PICO for this Meta-analysis and systematic review include: P: People diagnosed with an acute RTI, I: Consuming probiotic fermented dairy products (PFDP), C: Not consuming PFDP, O: Risk of incidence of URTIs or LRTIs. Also, articles were included for analysis if they conformed to the following criteria: (1) were RCT in design; (2) provided original data on the effect of probiotic fermented dairy products on incidence of respiratory tract infections; (3) were published in English; (4) done on human subjects; (5) had full text available, and (6) provided a comparison group. Trials that did not meet our inclusion criteria were excluded, and the remaining studies were selected for further analysis.

#### Data extraction

Data extraction was performed by two investigators, independently (SA and PJ) using a standardized data extraction sheet. Subsequently, full texts studies were assessed, and disagreements were resolved through discussion with a third independent researcher (BR). The following information was extracted: first author’s name, publication year, country/geographic location, study design including whether parallel or cross-over, target population, mean age, gender, number of participants, study duration, type and dosage of PFDP, relative risks (RR) and 95 % confidence interval (CI) of RTIs.

#### Quality assessment of studies

Two reviewers (MD & AD) independently assessed the quality of each study according to the Cochrane risk of bias [2], which is composed of the following criteria: random sequence generation, allocation concealment, blinding, and clarification of failures (imperfect outcome data), selective outcome reporting and other biases. According to the Cochrane guideline handbook, the words “yes,” “no,” and “unclear” corresponded to low, high, and unknown risk of bias, respectively. According to the mentioned domains, the overall quality of study was considered as good (low risk for all items), fair (low risk more than three items), and poor (low risk for 3 or fewer items).

#### Data synthesis and analysis

All analyses were performed using STATA software version 12 (STATA corp, College Station, TX, USA). Due to the fact that selected RCTs were carried out in different settings, a random-effects model was used to pool the RR and 95 %CI for outcomes following consumption of PFDP. Heterogeneity was examined using the I-squared (*I*^*2*^) index. An *I*^*2*^ value > 50 % was considered to indicate substantial heterogeneity between trials [4]. To explore the source of heterogeneity, in addition to the general analysis, we performed subgroup analyses by studied population, probiotic genus used in dairy products, type of dairy product, and type of RTI. Meta-regression analysis was also carried out to explore the effect of the duration of supplementation and age of participants of pooled estimates. The presence of publication bias was tested using the Egger’s regression asymmetry test and *P* < 0.05 was considered statistically significant, except where otherwise specified.

## Results

### Study characteristics

**Fig. 1:**
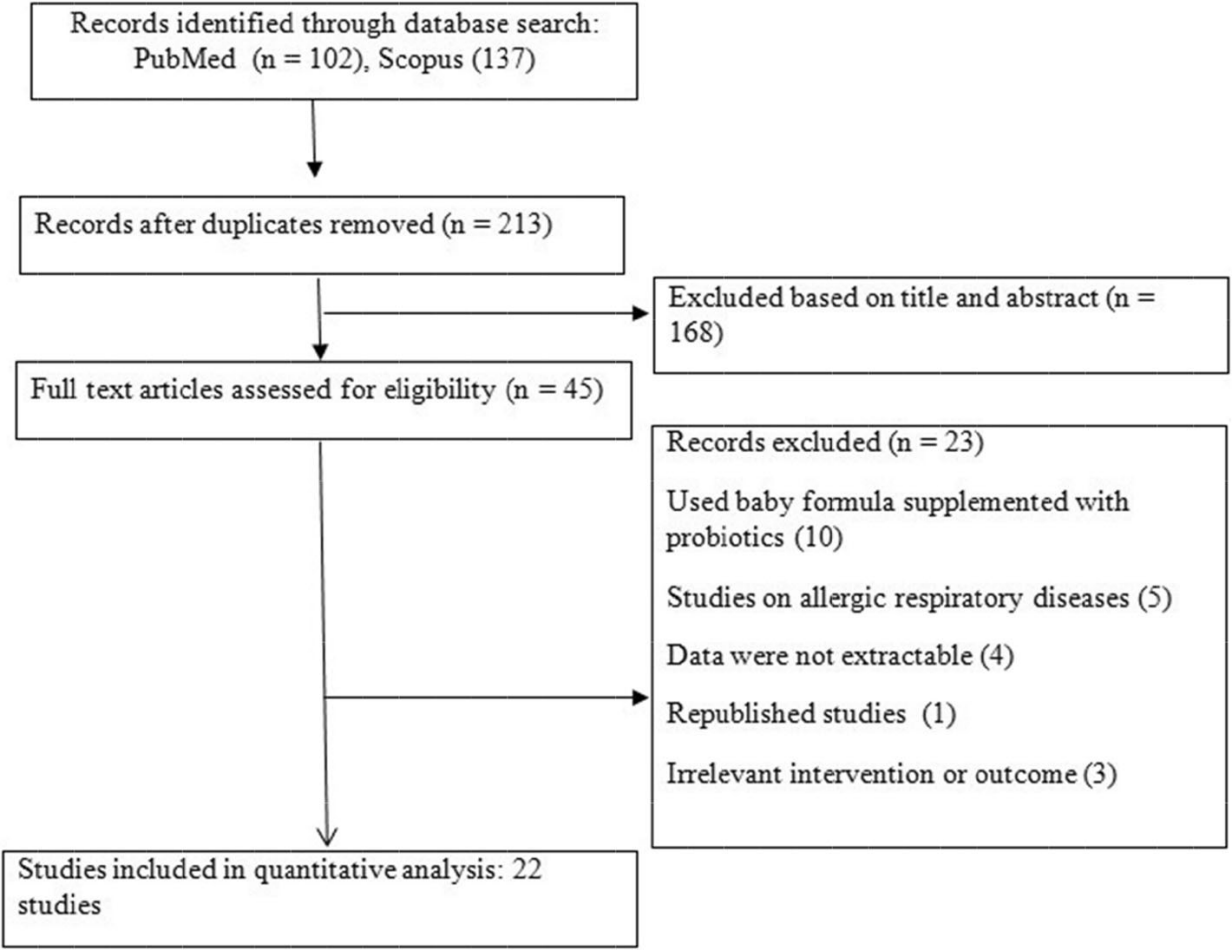
Flow diagram of the study

A total of 239 articles were identified through the systematic literature search of databases. After excluding 26 duplicate studies and removing 168 irrelevant publications based on titles/abstracts, 45 studies went under full-text screening. Of which, 23 paper were excluded based on the inclusion criteria because they used baby formula supplemented with probiotics as intervention, were on allergic respiratory diseases, were republished studies, did not report sufficient extractable data, or had irrelevant intervention or outcome. Finally, 22 clinical trials with 33 datasets [14, 26–46], with a total sample size of 10,190 subjects were included in this meta-analysis. The flow diagram of study selection is presented in Fig. 1. Some studies reported multiple results; we extracted all suitable data for such studies. For instance, the study by Agustina et al. [26] contained two interventions, a group received fermented milk with Lactobacillus casei CRL431 and another group received fermented milk with Lactobacillus reuteri DSM17938, which both were included. The study by Makino et al. [38] included two separate studies and both were eligible for our meta-analysis. Moreover, some studies reported results for different respiratory tract infections (RTIs) separately, which all effect sizes were included. Data on total RTIs, lower respiratory tract infections (LRTIs), and upper respiratory tract infections (URTIs) were reported in 5 studies with 6 data sets [26, 33, 36, 37, 45], 9 studies with 10 data sets [14, 29–32, 40, 42, 43, 46], and 14 studies with 17 data sets [14, 27–29, 31, 32, 34, 35, 38–41, 44, 46], respectively. Among the included studies, there were 10 studies with 17 data sets on children [14, 26, 27, 31–33, 36, 37, 40, 43] and 6 studies with 8 data sets for each adult [29, 34, 35, 39, 42, 44], and elderly population [28, 30, 38, 41, 45, 46]. The probiotic genus used in fermented dairy products was Lactobacillus in 20 studies [14, 26–38, 40–42, 44–46] and Bifidobacterium in 2 studies [39, 43]. Furthermore, the fermented dairy products used as intervention was milk in 14 studies with 21 data sets [26–28, 30–34, 36, 37, 40, 43–45], yogurt in 4 studies with 5 data sets [35, 38, 39, 41], and a dairy drink in 4 studies with 7 data sets [14, 29, 42, 46]. Concerning study design, all studies were parallel RCT, except for the study by Meng et al.[39], which had a crossover design. The sample size of the included studies ranged from 52 to 1104 participants and follow-up period was between 1 and 12 months. Based on the Cochrane scale, all included studies received scored as moderated to high quality. Other characteristics of the analyzed publications are reported in Table 1.

**Table 1:** Study characteristics

Study	Year	Country	Population	sex	Follow-up (month)	Intervention mean age (year ±sd)	Placebo mean age (year)	Intervention type	Intervention (n)	Placebo (n)	Outcomes
Agustina [26]	2012	Indonesia	Healthy children	Both	6	60.3 ± 13.7	58.9 ±14.2	Milk with 5.10^8^ CFU/day of Lactobacillus casei CRL431	120	126	Respiratory tract infections
								Milk with 5.10^8^ CFU/day of Lactobacillus reuteri DSM17938	124		
Hojsak [32, 33]	2010	Croatia	Healthy children	Both	3	4.32±1.52	4.46±1.45	Fermented milk with Lactobacillus GG at a dose of10^9^ CFU/ 100 ml of milk	139	142	Upper respiratory tract infections Lower respiratory tract infections Acute otitis media Pharyngitis Rhinitis Pneumonia
Hatakka [31]	2001	Finland	Healthy children	Both	7	4.6 ±1.5	4.4 ±1.5	Milk with 5­10 *x* 10^5 CFU/ml of strain Lactobacillus rhamnosus GG	282	289	Acute otitis media Sinusitis Acute bronchitis Pneumonia
Hojsak [32, 33]	2010	Croatia	hospitalized children	Both	NR	9.9±5.1	10.6± 5.0	Fermented milk with Lactobacillus GG at a dose of10^9^ CFU/ 100 ml of milk	376	366	Respiratory tract infections
Guo [30]	2018	China	Elderly patients with a single rib fracture	Both	1	57.2 ± 9.7	55.4 ± 7.8	Milk containing at least 6 × 10^9^ CFU Lactobacillus casei Shirota	102	102	Pneumonia
Guillemard [46]	2010	France	Free-living elderly volunteers	Both	3	76·0 ±5.92	76·0 ±5.18	Fermented dairy drink containing at least 10^10^ CFU/100 g of the probiotic Lactobacillus casei DN-114 001	537	535	Upper respiratory tract infections Lower respiratory tract infections
Guillemard [29]	2010	Germany	Healthy Shift Workers	Both	3	31.8 ±8.9	32.5 ±8.9	Fermented dairy drink containing at least 10^10^ CFU/100 g of the probiotic Lactobacillus casei DN-114 001	500	500	Upper respiratory tract infections Lower respiratory tract infections
Jespersen [34]	2015	Denmark	Healthy adult volunteers	Both	1.4	31.3 ± 10.57	31.6 ± 10.65	Milk drink containing 10^9^ CFU of L. casei 431	553	551	Common cold Influenza
Kumpu [36]	2012	Finland	Healthy children	Both	7	4.0 ±1.3	4.0 ±1.4	Milk with Lactobacillus rhamnosus GG ranged from 6.7 ×10^5^ to 1.9× 10^6^ CFU/ml	251	250	Respiratory tract infections
Corsello [27]	2017	Italy	Healthy children	Both	3	32.5 ± 9.7	33.7 ± 8.6	Milk Fermented containing 5.9 ×10^9^ Lactobacillus paracasei CBA L74	73	73	Upper respiratory tract infections
Kinoshita [35]	2019	Japan	Women healthcare workers	Female	4	39.3 ±11.5	39.4 ±11.4	Yogurt drink fermented with Lactobacillus delbrueckii ssp. bulgaricus OLL1073R-1 at a dose of 1.12× 10^9^ CFU	479	482	Influenza
Makino [38]	2010	Japan	Healthy elderly individuals	Both	3	66.7 ± 6.25	39.4 ±11.4	yoghurt fermented with Lactobacillus delbrueckii ssp. bulgaricus OLL1073R-1 and Streptococcus thermophilus OLS3059 at a dose of 2·0–3·5 × 10^8^ and 6·3–8·8 × 10^8^ CFU/g, respectively	44	43	Common cold
					2	74.5 ± 2.75	39.4 ±11.4	yoghurt fermented with Lactobacillus delbrueckii ssp. bulgaricus OLL1073R-1 and Streptococcus thermophilus OLS3059 at a dose of 2·0–3·5 × 10^8^ and 6·3–8·8 × 10^8^ CFU/g, respectively	30	30	Common cold
Prodeus [40]	2016	Russia	Healthy children	Both	3	4 ± 1	4 ± 1	Fermented milk product containing L casei CNCM I-1518 at a dose of 10^10^ CFU/100 g	300	299	Upper respiratory tract infections Lower respiratory tract infections Rhinopharyngitis
Pu [41]	2017	China	Healthy elderly	Both	3	57.39±8.47	59.54±8.08	Yogurt supplemented with a probiotic strain, Lactobacillus paracasei N1115 at a dose of 3.6×10^7^ CFU/mL	115	118	Upper respiratory tract infections
Mai [37]	2020	Vietnam	Healthy children	Both	3	4.3±0.83	4.5±0.71	Fermented milk containing 10^8^ CFU/mL of Lactobacillus casei strain Shirota	510	493	Respiratory tract infections
Meng [39]	2016	USA	Healthy adults	Both	1	28.0 ± 1.2	4.5±0.71	Fermented yogurt with Bifidobacterium animalis subsp. lactis BB-12 at a dose of 10 ± 0.5 CFUs/day	26	26	cold/flu infection
Merenstein [14]	2010	USA	Healthy children	Both	3	4.86 ±1.12	4.94 ±1.13	Fermented probiotic dairy drink containing the probiotic strain Lactobacillus paracasei subsp. Paracasei at a dose of 1 ×10^8^ CFU/g	314	324	Upper respiratory tract infections Lower respiratory tract infections
Sazawal [43]	2010	India	Healthy children	Both	12	1.85 ± 0.53	1.90 ± 0.56	Milk fortified with 2.4 g/day of prebiotic oligosaccharide and 1.9 ×10^7^ CFU /day of probiotic Bifidobacterium lactis HN019	312	312	Pneumonia Lower respiratory tract infections
Rongrungruang [42]	2015	Thailand	Adult hospitalized patients in medical wards	Both	3	73.09±13.16	68.95±18.45	Fermented dairy product containing 8x10^9^ CFU Lactobacillus casei (Shirota strain)	75	75	Pneumonia
Shida [44]	2017	Japan	Healthy middle‑aged office workers	Male	3	40.6 ±5.3	40.5 ±5.9	Fermented milk with Lactobacillus casei strain Shirota at a dose of 1.0 × 10^11^ CFU	49	47	Upper respiratory tract infections Common cold Influenza
Fujita [28]	2013	Japan	Elderly persons	Both	5	83.2 ± 9.1	83.5 ± 8.9	Fermented milk containing Lactobacillus casei strain Shirota at a dose of 4 × 10^10^ CFU	76	78	Upper respiratory tract infections
Puyenbroeck [45]	2012	Belgium	Elderly persons	Both	5.8	83.95 ± 9.24	84.17 ± 11.5	Fermented milk that contained 6.5 × 10^9^ live Lactobacillus casei Shirota	375	362	Respiratory tract infections

*CFU* Colony forming unit, *sd* standard deviation

### Quantitative analysis

**Fig. 2:**
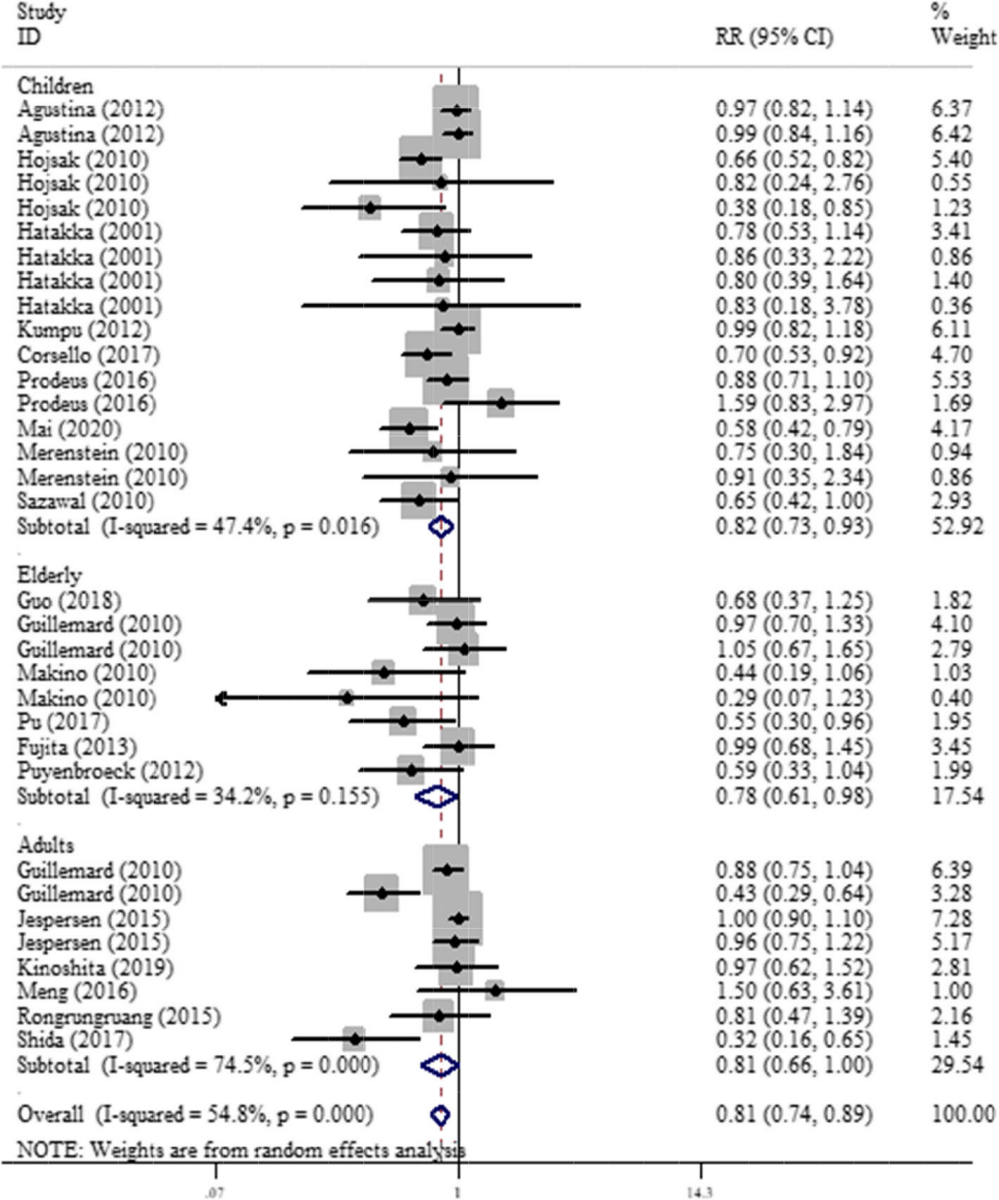
Meta-analysis for the effect of fermented probiotic dairy products on respiratory tract infections stratified by studied population

Overall and stratified analysis by studied population for the effect of probiotic fermented dairy products (PFDP) on RTIs is presented in Fig. 2. When all studies were pooled, it was found that, compared with placebo, consumption of PFDP has a significant protective effect against RTIs in the overall analysis (RR = 0.81, 95 %CI: 0.74 to 0.89) and in children (RR = 0.82, 95 %CI: 0.73 to 0.93), adults (RR = 0.81, 95 %CI: 0.66 to 1.00), and elderly population (RR = 0.78, 95 %CI: 0.61 to 0.98), with a significant heterogeneity across studies (I2 = 54.8 %, *P* < 0.001). The significant decreased risk of RTIs was also observed for URTIs (RR = 0.83, 95 %CI: 0.73 to 0.93), while, this association was marginal for LRTIs (RR = 0.78, 95 %CI: 0.60 to 1.01, *P* = 0.06). In the subgroup analysis, the significant impact of PFDP on RTIs was modified by probiotic genus and type of dairy product used for intervention; while, PFDP consumption had a protective effect on RTIs when Lactobacillus and milk were used as probiotic and fermented dairy product, respectively, but no significant effect was found in studies which administered Bifidobacterium and dairy drink or yogurt (Table 2).

**Table 2:** Subgroup analyses for the effect of probiotic dairy products on respiratory tract infections

Subgrouped by		No. of data sets		RR^a^ (95 % CI)	*P* value^b^		I2 (%)^c^		*P* value^d^
**Overall**		33	0.81 (0.74 to 0.89 )			˂0.001	54.8	˂0.001	
**Population type**									
Children		17	0.82 (0.73 to 0.93 )			0.001	47.4	0.01	
adults		8	0.81 (0.66 to 1.00 )			0.04	41.8	0.07	
Elderly		8	0.78 (0.61 to 0.98 )			0.03	34.2	0.15	
**Probiotic genus**									
Lactobacillus		31	0.81 (0.74 to 0.90 )			˂0.001	55.6	˂0.001	
Bifidobacterium		2	0.90 (0.41 to 2.01 )			0.80	64.6	0.09	
**Dairy type**									
Milk		21	0.83 (0.74 to 0.92 )			˂0.001	58.0	˂0.001	
Dairy drink		7	0.80 (0.64 to 1.02 )			0.07	54.3	0.04	
Yoghurt		5	0.71 (0.44 to 1.13 )			0.15	49.6	0.09	
**Type of infection**									
RTIs		6	0.82 (0.67 to 1.00 )			0.05	71.1	0.004	
URTIs		`17	0.83 (0.73 to 0.93 )			0.002	52.3	0.006	
LRTIs		10	0.78 (0.60 to 1.01 )			0.06	43.3	0.07	

*RTIs* Respiratory tract infections, *URTIs *Upper respiratory tract infections, *LRTIs *Lower respiratory tract infections, *RR* relative risk

^a^Effect size was expressed as relative risk and 95% confidence interval

^b^For meta-analysis: *P* ≤ 0.05 was considered to be a significant effect by using a random-effects model

^c^The I^2^ statistic was calculated by using Cochran’s test, and I^2^  statistic > 50% was considered to indicate significant heterogeneity across studies

^d^*P* value for I2

### Fermented probiotic dairy products and specific respiratory tract infections

**Fig. 3:**
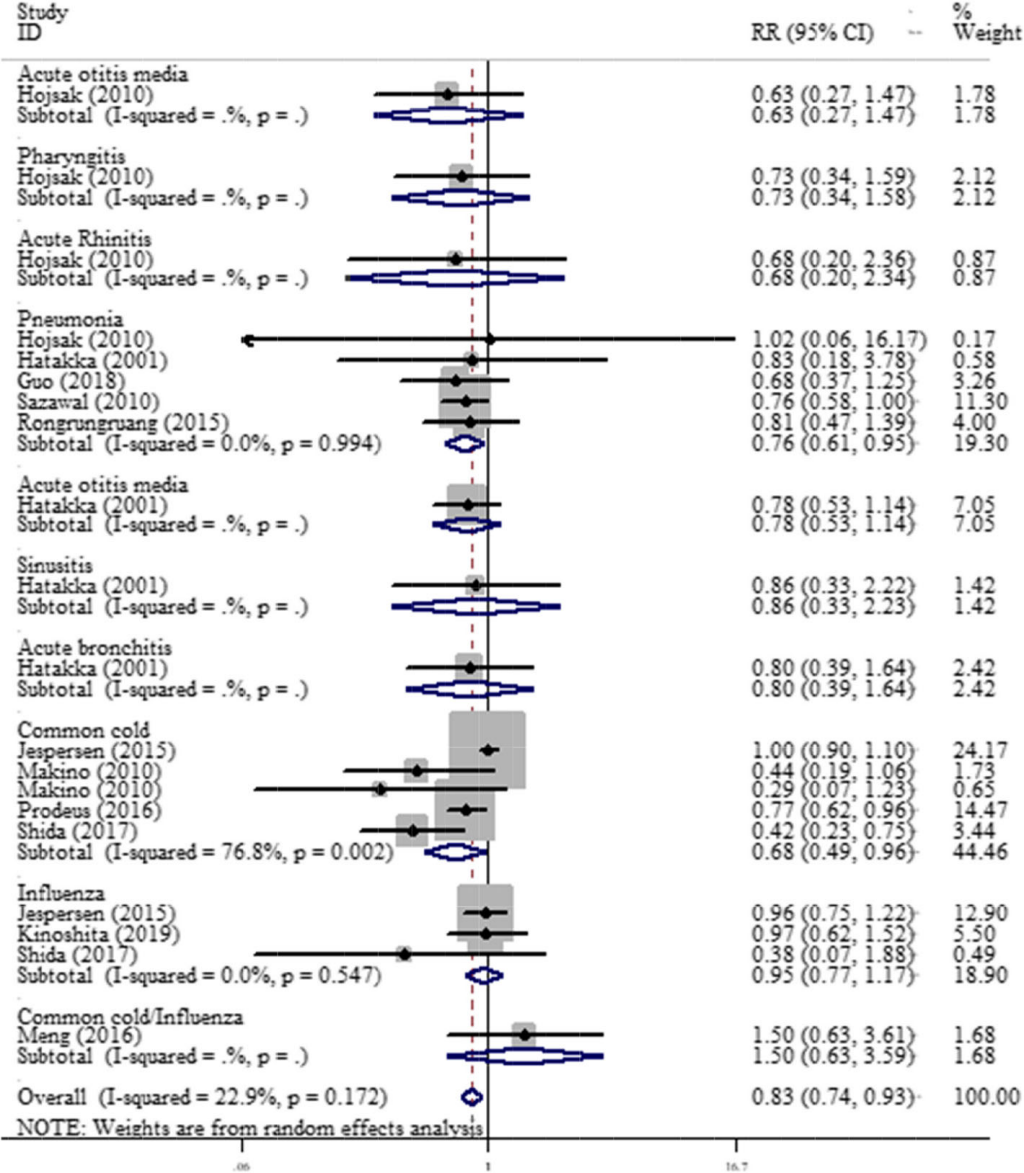
Meta-analysis for the effect of fermented probiotic dairy products on specific respiratory tract infections

Meta-analysis for the effect of PFDP on specific respiratory tract infections showed that the consumption of PFDP has a protective effect on pneumonia (RR = 0.76, 95 %CI: 0.61 to 0.95) and common cold (RR = 0.68, 95 %CI: 0.49 to 0.96). PFDP had no significant effect on other RTIs (Fig. 3).

### Meta-regression, sensitivity analysis, and publication bias

**Fig. 4:**
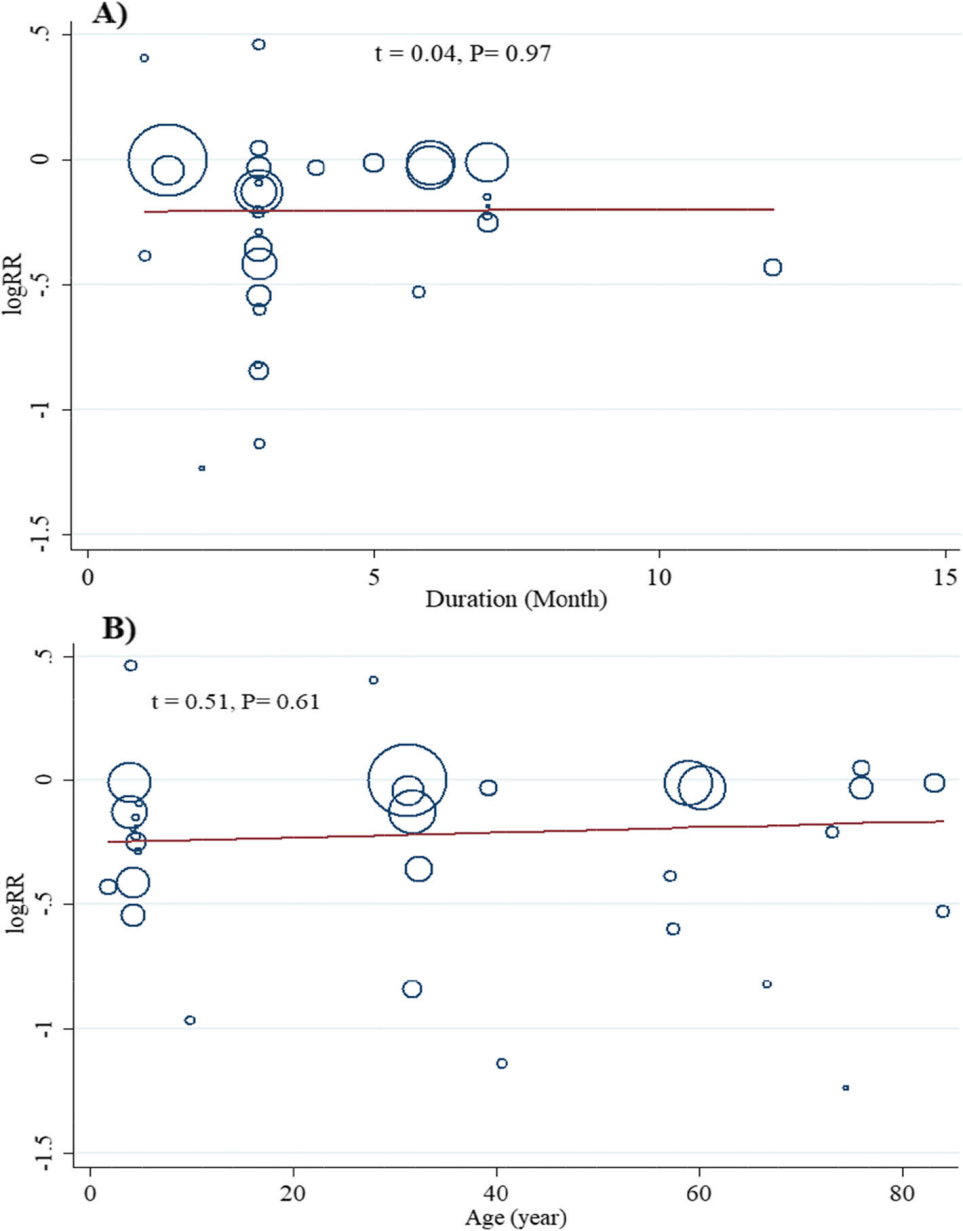
Meta-regression analysis for the effect of fermented probiotic dairy products on respiratory tract infections based on follow-up duration (**A**) and age of participants (**B**)

**Fig. 5:**
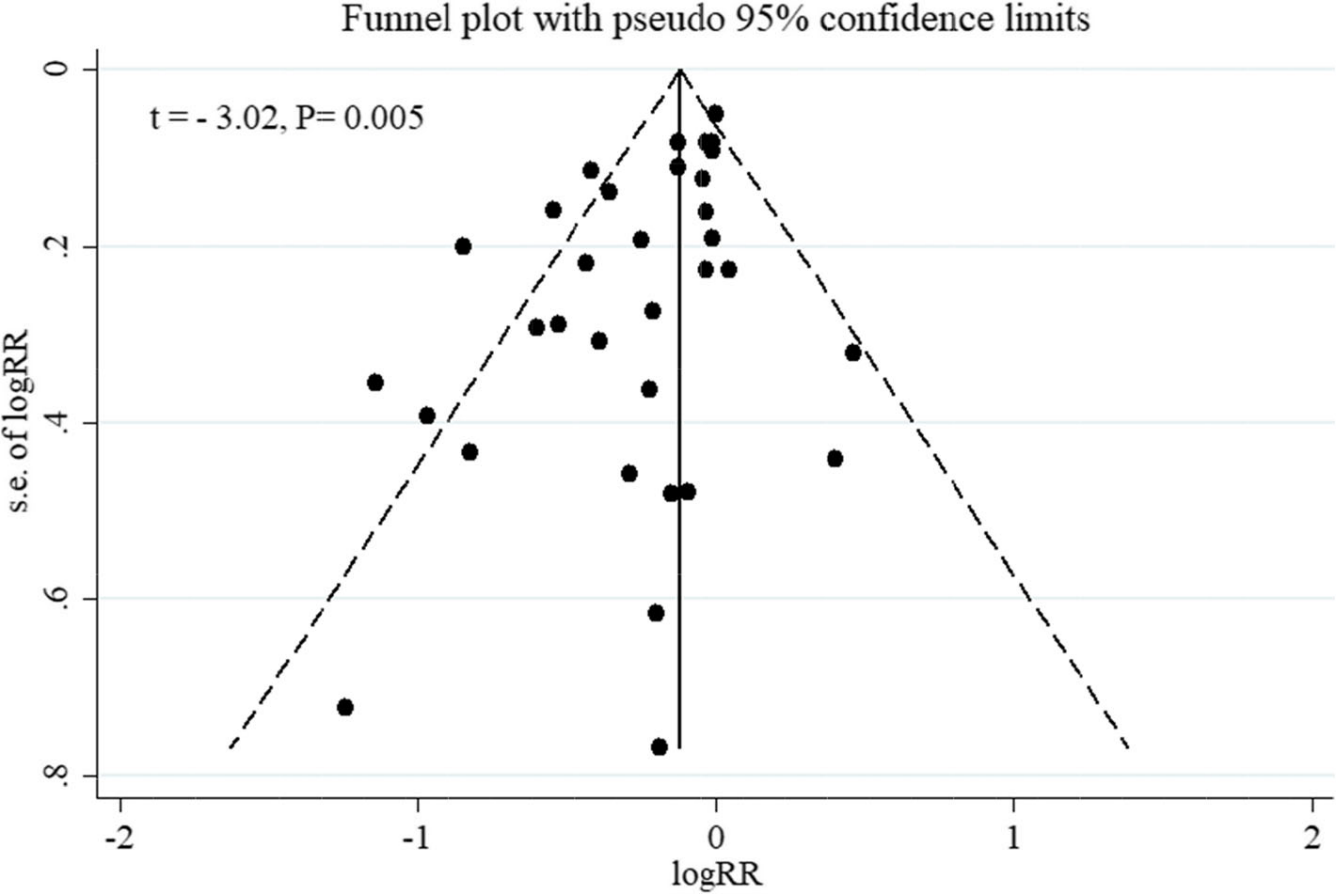
Funnel plot for publication bias in studies investigating the effect of fermented probiotic dairy products on respiratory tract infections

Meta-regression analysis showed that the effect of PFDP on RTIs was not modified by the duration of supplementation and age of participants (Fig. 4). There was a significant evidence for possible publication bias based on funnel plots asymmetry and Egger’s linear regression test (t = − 3.02, *P* = 0.005) (Fig. 5). In the sensitivity analysis by removing one study at a time and reanalyzing other studies, the polled effect size ranged from (RR = 0.79, 95 %CI: 0.72 to 0.88) to (RR = 0.83, 95 %CI: 0.76 to 0.91) and no single study significantly affected the pooled effect estimate, showing the reliability of the findings.

## Discussion

During the past years, numerous investigations have evaluated the potential role of fermented probiotic dairy products against RTIs. Notwithstanding, these studies yielded inconclusive findings. Differences in the experimental design, lower sample sizes, and bacterial strains used in the preparation of the fermented products might be the underlying cause of such conflicting results. To resolve the problem of inconsistency by abrogating the limiting issues present in the individual studies we conducted the current meta-analysis which is the most up-to-date study that contained a significantly higher frequency of studies and individuals in the intervention/placebo groups, and indicated beneficial effects of FPDPs supplementation in reducing the risk of RTIs. Accordingly, overall and stratified analysis highlighted decreased risk of RTIs in overall population, all age subgroup, dairy products fermented with Lactobacillus and those who consume fermented probiotic milk, but not diary drink and yogurt.

Probiotics are defined as “beneficial live microorganisms which its administration in optimal amount confer a health benefit to the user”. The boosting effects of probiotics on the function of gastrointestinal and respiratory systems have been proposed by several studies performed on humans and animals [47, 48]. However, the observed beneficial effects are bacterial strain dependent. It is highlighted that lactobacillus casei resistant to gastric acid and bile. In this regard, several studies have shown that lactobacillus-containing products reach the lower digestive tract without losing its activity, and after localization balance the intestinal flora by promotion of immune cells that are produced in the lower intestinal tract. These cells might migrate to other mucosal sites and contribute in protection against pathogens [49–51].

So far, several mechanisms have been suggested on the effectiveness of probiotics to promote immune system. Firstly, Lactobacillus cause an anti-inflammatory impact by reduction of Interleukin-12 (IL-12) and stimulation of Interleukin-10 (IL-10) [52]. From immunological point of view, IL-10 derived from CD4 + T-helper type 2. This cytokine identified as potent inhibitor of monocyte/macrophage function and suppress the production of many pro-inflammatory cytokines [53]. Secondly, probiotics present an immunostimulatory effect which resulted in activation of innate and acquired immunity cells and subsequently production of innate and acquired immunity peptides. Paneth cells, neutrophils, and epithelial cells are among activated cells which produce antimicrobial peptides (AMPs) like lysozyme, lactoferrin, defensins and defend the body against pathogens [54, 55]. Moreover, secretory immunoglobulin A (IgA) which is a functional acquired immunity peptides defenses against pathogens either by immune exclusion or neutralization mechanism [56]. Studies have shown that probiotics induce AMPs, IgA, and IgG, resulting in an augmentation of immune system against infections [57, 58]. In confirm of the aforementioned theoretical facts, Reale et al. showed that probiotic intake can restore natural killer (NK) cell activity, member of innate immunity cells, which strengthen the host’s immune defense and induce a quick recovery by shortening the duration of infection [59, 60]. As our results show, consumption of FPDPs significantly decreased risk of RTIs in all age groups which can be a promising finding supported by potential biological mechanisms. Reduction of RTIs by FPDPs is highly important is it is accompanied by a reduction in medication use, working and school days loss, and social burden. It should be considered that the non-significant effect of studies which administered Bifidobacterium and dairy drink or yogurt on RTIs is due to small number of analyzed studies in these subgroups, which is important to be interpreted with a high caution.

Our meta-analysis was not bereft of limitations and caveats. First, we searched only English-written papers, which may raise the possibility of omission of potentially valuable studies and cause publication bias. Second, we observed a significant heterogeneity among the studies that might stem largely from, ethnicity of participants, year of publication, age, clinical heterogeneity, unreported and unknown study characteristics and many other factors which we are not able to attenuate their impact on final analysis. Therefore, for finding any sources of heterogeneity and attenuating their effects, we conducted subgroup analysis and weighted meta-regression. Collectively, the results of meta-regression showed that duration of supplementation and age of participants were not the expected source of heterogeneity, but probiotic genus and type of dairy product used for intervention were found as sources of observed heterogeneity. However, to deal with statistical heterogeneity, a random-effects model was applied for analyzes, which typically produces more conservative estimates of the significance of a result (a wider confidence interval), as it gives proportionately higher weights to smaller studies and lower weights to larger studies than fixed effect analysis.

## Conclusions

Considering all the facts, this was the first comprehensive systematic review and meta-analysis of the effect of probiotic dairy products on respiratory tract infections, by including 22 clinical trials with 33 datasets. Our analysis indicated protection effect of FPDPs against RTIs in all age subgroup.

## Supplementary Information

**Additional file 1: Supplemental file 1.** Search strategy of the study. **Supplemental Table 1.** Sensitivity analysis by removing one study at a time and reanalyzing other studies

## Data Availability

The datasets used and/or analysed during the current study are available from the corresponding author on reasonable request.

## Abbreviations

PFDP: Probiotic fermented dairy products
RTIs: Respiratory tract infections
RCTs: Randomized controlled trials
URTIs: Upper respiratory tract infections
LRTIs: Lower respiratory tract infections
RR: Relative risks
CI: Confidence intervals
IL-12: Interleukin-12
IL-10: Interleukin-10
AMPs: Antimicrobial peptides
IgA: Immunoglobulin A
IgG: Immunoglobulin G
NK: Natural killer
