# Supplementary material for: Effect of probiotic fermented dairy products on incidence of respiratory tract infections: a systematic review and meta-analysis of randomized clinical trials
Source: Nutr J. 2021 Jun 28;20:61. doi: 10.1186/s12937-021-00718-0 (PMC8240278; doi:10.1186/s12937-021-00718-0)
Supplement: Supplementary file 1 — Additional file 1: Supplemental file 1. Search strategy of the study. Supplemental Table 1. Sensitivity analysis by removing one study at a time and reanalyzing other studies [file 12937_2021_718_MOESM1_ESM.docx]

Supplemental file 1. Search strategy of the study:

**((((((((((((((((((((((((("Dairy Products"[Mesh]) OR (Dairy[Title/Abstract])) OR (Dairies[Title/Abstract])) OR ("Milk"[Mesh])) OR (Milk[Title/Abstract])) OR ("Cheese"[Mesh])) OR (Cheese[Title/Abstract])) OR ("Yogurt"[Majr])) OR (yogurt[Title/Abstract])) OR (yoghurt[Title/Abstract])) OR (yoghourt[Title/Abstract])) OR (Kefir[Title/Abstract])) OR (Curd[Title/Abstract])) OR (buttermilk[Title/Abstract])) OR (lassi[Title/Abstract])) OR (doogh[Title/Abstract])) OR (dahi[Title/Abstract])) OR (amasi[Title/Abstract])) OR (filmjolk[Title/Abstract])) OR (chal[Title/Abstract])) AND (((fermented[Title/Abstract]) OR (cultured[Title/Abstract])) OR ((((((((("Probiotics"[Majr]) OR ("Synbiotics"[Majr])) OR ("Prebiotics"[Majr])) OR (Probiotics[Title/Abstract])) OR (Probiotic[Title/Abstract])) OR (Synbiotics[Title/Abstract])) OR (Synbiotic[Title/Abstract])) OR (Prebiotics[Title/Abstract])) OR (Prebiotic[Title/Abstract])))) AND (english[Filter])) AND (((((((("Randomized controlled trial"[Title/Abstract]) OR ("Controlled Clinical Trial"[Title/Abstract])) OR (Randomized’’[Title/Abstract])) OR (Randomly[Title/Abstract])) OR (Placebo[Title/Abstract])) OR (Trial[Title/Abstract])) OR (Groups[Title/Abstract])) AND (english[Filter])) NOT ((((((rat[Title/Abstract]) OR (rats[Title/Abstract])) OR (animal[Title/Abstract])) OR (animals[Title/Abstract])) OR (mice[Title/Abstract])) OR (Mouse[Title/Abstract]) AND (english[Filter])) AND (english[Filter]))) AND ((((((((((((((((((("Respiratory Tract Infections"[Majr]) OR ("Respiratory Tract Infections"[Title/Abstract])) OR (Respiratory[Title/Abstract])) OR (cold[Title/Abstract])) OR (laryngitis[Title/Abstract])) OR (sinusitis[Title/Abstract])) OR (pharyngitis[Title/Abstract])) OR (tonsillitis[Title/Abstract])) OR (rhinitis[Title/Abstract])) OR (rhinosinusitis[Title/Abstract])) OR (otitis[Title/Abstract])) OR (influenza[Title/Abstract])) OR (Epiglottitis[Title/Abstract])) OR (Laryngotracheitis[Title/Abstract])) OR (bronchitis[Title/Abstract])) OR (bronchiolitis[Title/Abstract])) OR (tracheitis[Title/Abstract])) OR (pneumonia[Title/Abstract])) OR (Rhinopharyngitis[Title/Abstract]) AND (english[Filter]))**

Supplemental Table 1. Sensitivity analysis by removing one study at a time and reanalyzing other studies

|  |  | 95% confidence interval | |
| --- | --- | --- | --- |
| Study omitted | Relative risk | lower  limit | upper limit |
| Agustina (2012)  Agustina (2012)  Hojsak (2010)  Hojsak (2010)  Hojsak (2010)  Hatakka (2001)  Hatakka (2001)  Hatakka (2001)  Hatakka (2001)  Guo (2018)  Guillemard (2010)  Guillemard (2010)  Guillemard (2010)  Guillemard (2010)  Jespersen (2015)  Jespersen (2015)  Kumpu (2012)  Corsello (2017)  Kinoshita (2019)  Makino (2010)  Makino (2010)  Prodeus (2016)  Prodeus (2016)  Pu (2017)  Mai (2020)  Meng (2016)  Merenstein (2010) .  Merenstein (2010)  Sazawal (2010)  Rongrungruang (2015)  Shida (2017)  Fujita (2013)  Puyenbroeck (2012) | 0.80216944  0.80131823  0.82649708  0.81342727  0.82371992  0.81446469  0.8130284  0.81360143  0.81341606  0.81645924  0.8068583  0.80749667  0.80654532  0.83948594  0.79962415  0.805255  0.80209231  0.82038909  0.80907106  0.82009894  0.8182292  0.80825585  0.80614495  0.82117641  0.82951856  0.8092286  0.81412339  0.81261992  0.81984699  0.81339222  0.8292737  0.8074736  0.81975716 | 0.72623622  0.72574359  0.75266945  0.74008882  0.75152761  0.73982596  0.73958224  0.73989528  0.74015534  0.74262404  0.73242927  0.73390049  0.72948635  0.76991522  0.72422868  0.73021156  0.72678638  0.74562579  0.73512155  0.74723822  0.74568999  0.73233235  0.73459613  0.74782056  0.7566492  0.7367627  0.74060541  0.73920822  0.74578714  0.73935145  0.75848103  0.73340511  0.74616545 | 0.88604194  0.88476276  0.90756631  0.89403313  0.90284699  0.89663345  0.89376831  0.89464992  0.89392811  0.8976354  0.88885081  0.88847315  0.89174438  0.91534317  0.88286865  0.88801062  0.8852011  0.90264887  0.8904596  0.90006399  0.89782488  0.89205068  0.88466263  0.90172791  0.90940559  0.88882196  0.89493924  0. 89332217  0.90126127  0.89484769  0.90667379  0.88902253  0.90060693 |
| Combined | 0.81409043 | 0.74141449 | 0.89389031 |
